# Intravenous ketamine for depression: A clinical discussion reconsidering best practices in acute hypertension management

**DOI:** 10.3389/fpsyt.2022.1017504

**Published:** 2022-09-29

**Authors:** Ryan Yip, Jennifer Swainson, Atul Khullar, Roger S. McIntyre, Kevin Skoblenick

**Affiliations:** ^1^Department of Emergency Medicine, University of Alberta, Edmonton, AB, Canada; ^2^Department of Psychiatry, University of Alberta, Edmonton, AB, Canada; ^3^Misericordia Community Hospital, Edmonton, AB, Canada; ^4^Northern Alberta Sleep Clinic, Edmonton, AB, Canada; ^5^Grey Nuns Community Hospital, Edmonton, AB, Canada; ^6^Mood Disorders Psychopharmacology Unit, University Health Network, Toronto, ON, Canada; ^7^Department of Psychiatry, University of Toronto, Toronto, ON, Canada; ^8^Department of Pharmacology, University of Toronto, Toronto, ON, Canada; ^9^Brain and Cognition Discovery Foundation, Toronto, ON, Canada; ^10^Royal Alexandra Hospital, Edmonton, AB, Canada; ^11^Neuroscience and Mental Health Institute, University of Alberta, Edmonton, AB, Canada

**Keywords:** ketamine, hypertension, hypertensive emergency, ketamine infusion, treatment resistant depression

## Abstract

Ketamine is a versatile medication with an emerging role for the treatment of numerous psychiatric conditions, including treatment resistant depression. Current psychiatry guidelines for its intravenous administration to treat depression recommend regular blood pressure monitoring and an aggressive approach to potential transient hypertensive episodes induced by ketamine infusions. While this approach is aimed at ensuring patient safety, it should be updated to align with best practice guidelines in the management of hypertension. This review defines and summarizes the currently recommended approach to the hypertensive emergency, the asymptomatic hypertensive urgency, and discusses their relevance to intravenous ketamine therapy. With an updated protocol informed by these best practice guidelines, ketamine treatment for depression may be more accessible to facilitate psychiatric treatment.

## Introduction

Ketamine is versatile as both an analgesic and sedating medication. At the lower dosing range, ketamine is an adjunctive pain medication to acetaminophen, non-steroidal anti-inflammatory drugs, and opioids. Ketamine also provides analgesia and sedation for prolonged procedures such as fracture reduction or chest tube insertion. In higher doses of 1–1.5 mg/kg ketamine is used for induction of general anesthesia in both the emergency department and operating room settings. In addition to these various applications, interest is growing in the use of ketamine for its utility in treating psychiatric disorders.

Intravenous ketamine delivered in sub-anesthetic doses (0.5–1.0 mg/kg) has shown effectiveness in reducing depressive symptoms and suicidal ideation in both unipolar and bipolar depression (1–3). Consensus papers from Canada (1), the United States (4), and internationally (5) have recognized its emerging role in the treatment of depression. Unfortunately, administering intravenous ketamine demands significant resources. Monitoring is required due to the potential adverse effects, one of which is a transient episode of hypertension.

Additionally, ketamine has also been used in psychiatric research settings, extensively employed as a model for schizophrenia in humans (6) and non-human primates alike (7). For these studies, ketamine is usually given either as a bolus followed by an infusion or simply a bolus *via* intravenous administration at a sub-anesthetic dose (0.1–0.7 mg/kg) to healthy volunteers (8). Due to the known effect of ketamine to transiently increase blood pressure, studies often mention they record blood pressure but it may not be reported (9–11). When it is reported, the blood pressure changes have been low – reported as transiently increased SBP in one study (mean 18.6 +/- 2.6 mmHg) (12) and transiently increased SBP of ~20 mmHg in another (13). When compared to the mean SBP elevation of 57 mmHg observed during 9 min of simple exercise in healthy volunteers (14), these small and brief increases caused by ketamine are trivial. As such, while monitoring blood pressure in these healthy volunteers seems to report insignificant transient changes, the recommendations regarding blood pressure monitoring in a research setting are a matter of discourse for the research protocols and ethics committees reviewing them. Clinical studies on IV ketamine for depression are subject to similar ethics boards requirements, and it is important to distinguish the necessity of monitoring for ethical purposes in a research setting vs. clinical need and utility in a clinical setting. This paper reviews the minimal risk of serious complications related to blood pressure in a therapeutic application.

The Canadian Network for Mood and Anxiety Disorders (CANMAT) task force statement on the use of ketamine for depression recommends that blood pressure should be monitored before and during ketamine infusions and for at least 1 h post infusion. In addition, it is recommended that patients with baseline blood pressures <140/90 mmHg should not proceed with treatment (1). An international consensus paper on ketamine treatment also suggests that individuals with other conditions such as uncontrolled hypertension, central aneurysmal disease, significant valvular disease, or New York Heart Association Class III failure should also be excluded from treatment (5). Furthermore, the CANMAT task force statement recommends a blood pressure level of 160/100 mmHg as the threshold to pause infusion and resume treatment when blood pressure falls below 160/100 mmHg, with or without antihypertensive treatment. The concern is that these elevations will cause a complication of elevated blood pressure, the hypertensive emergency. Where blood pressure is a cause for concern, it has been suggested that it may be treated with beta-blockers, alpha agonists, vasodilators, or calcium channel blockers (15).

Based on the recommendations, a practitioner hoping to administer intravenous ketamine for TRD must involve the availability of personnel trained in resuscitation, airway support, and advanced cardiac life support. In addition, uncontrolled hypertension and white coat hypertension in the general patient population is not uncommon. Based on above criteria these patients may not be eligible for ketamine therapy. Data from clinical trials shows that this threshold for pausing infusion is crossed not infrequently. This suggests that applying these parameters to a real-world population would mean that a number of patients would not receive a complete treatment session, or would receive antihypertensive treatment before resuming. Clearly, there are multiple barriers to the routine administration of intravenous ketamine therapy and it is unclear what level of concern is necessary regarding hypertension associated with treatment.

Hypertension is a very common co-morbidity in the emergency department patient (16). The management of asymptomatic hypertension and hypertensive emergencies are an essential part of an emergency physician's skillset. With this experience, emergency medicine (EM) is uniquely well-positioned to provide an objective perspective on the level of concern for blood pressure effects of intravenous ketamine therapy as well as providing guidance for intervention. When comparing the existing literature for hypertensive management in the emergency department to hypertension as an adverse effect of intravenous ketamine therapy, the current recommendations for blood pressure monitoring and treatment from consensus statements in psychiatry should be revisited to align with standards of practice in management of transient hypertension in the emergency setting.

## Subsections

### Blood pressure elevations in the emergency room setting

The approach to hypertension in the emergency room requires classifying hypertensive patients into those with asymptomatic elevated blood pressure and those in hypertensive emergency. The true hypertensive emergency warrants acute blood pressure reduction and specialist consultation for further workup and management.

A hypertensive emergency is defined as moderate to severe hypertension (systolic blood pressure (SBP) ≥ 180 mmHg, diastolic blood pressure (DBP) ≥ 110 mmHg) with evidence of end organ dysfunction (EOD), most commonly involving the heart, brain, or kidneys. Specifically, emergency physicians look for signs and symptoms of acute stroke, cardiac ischemia, pulmonary edema, encephalopathy, and congestive heart failure (17). Although frequently accompanied by an elevated blood pressure, symptoms such as headache, epistaxis, and dizziness are not evidence of EOD (Table 1). These symptoms in isolation with an elevated blood pressure, do not constitute a hypertensive emergency nor do they indicate the need for acute blood pressure reduction (17, 18). In contrast to this, patients with hypertensive emergencies are treated with anti-hypertensives such as beta-blockers, calcium channel blockers, and vasodilators. The goal is a maximal reduction of mean arterial pressure by 20–25% within the 1st h and a target blood pressure of 160/100 mmHg by 2–6 h (17).

**Table 1:** Signs or symptoms of hypertensive emergencies, hypertension, and common adverse effects of ketamine.

**Signs/Symptoms of hypertensive emergency**	**Non-emergent hypertensive signs/symptoms**	**Common adverse effects of ketamine**
• Crushing chest pain or pressure • Decreased (not altered) level of consciousness • Severe abdominal pain • Shortness of breath • Syncope	• Headache • Dizziness • Epistaxis	• Dissociation • Anxiety • Headache • Dizziness • Nausea/vomiting • Blurred vision

Only the signs and symptoms of hypertensive emergencies would prompt a blood pressure check.

Outside of a true hypertensive emergency, patients may have elevated blood pressures with or without a history of pre-existing hypertension. In cases where patients present with asymptomatic elevated blood pressure, there is no indication for acute blood pressure lowering. This is supported by the American College of Emergency Physicians policy statement on asymptomatic elevated blood pressure, which recommends that routine emergency department investigations and medical intervention is not required for patients with asymptomatic markedly elevated blood pressure (18). Treating asymptomatic hypertension acutely does not have identified benefits, even in patients with many months of untreated hypertension. A large retrospective cohort study of primary care patients meeting criteria for moderate to severe hypertension (SBP ≥ 180 mmHg, DBP ≥ 110 mmHg) that went untreated found that rates of major adverse cardiovascular events were extremely low. Specifically, events including acute coronary syndrome, stroke, transient ischemic attack, uncontrolled hypertension (≥140/90 mmHg), and hospital admission occurred in >1% of patients at 7 days, 1 month, and 6 months after diagnosis (19). Clearly, even for asymptomatic hypertensive individuals the hypertensive emergency is extremely rare - even after 6 months of untreated hypertension.

### Ketamine in treatment-resistant depression—cardiovascular effects

The adverse effects of intravenous ketamine infusions for treatment-resistant depression (TRD) are well-characterized, most commonly including dissociative effects and transient hypertension. Other common, also transient effects include anxiety, blurred vision, dizziness, headache, and nausea or vomiting (15, 20). Most of these side effects were only mild or moderate, well tolerated, and transient, with all of them ceasing within 4 h post-administration of short-term ketamine for TRD (21, 22).

In randomized trials, typically involving administration of a single dose of intravenous ketamine, transient hemodynamic effects have been observed including increases in heart rate and blood pressure (21). This hypertensive effect may be blunted in the oral formulation of ketamine, however it comes at the cost of potentially lowered efficacy (23). Ketamine's receptor binding profile is quite diverse with at least 8 different cellular receptor systems involved (24). While ketamine's psychoactive effects are primarily attributed to its NMDA antagonism (25), its effects on heart rate and blood pressure have been attributed to a stimulation of the sympathetic nervous system (26) and increased catecholamine release (27). Early experiments have shown that ketamine-induced transient hypertension and tachycardia can be blunted by a co-administration of diazepam (28), a benzodiazepine acting as a positive allosteric modulator of the GABA receptor (29) and a common agent used to counteract sympathomimetic toxicity (30).

Increases in SBP and DBP begin shortly after administration and peak at around 30–50 min with SBP and DBP increases from 10 to 50% above pre-dose values. Increases in blood pressure and heart rate are usually mild and transient, resolving at ~2–4 h after dose administration, with no serious or persistent cardiovascular events reported (1, 21).

A relatively large case series involving 66 participants with 684 total infusions reported that blood pressure peaked at 30 min after infusion starts and only 9% of infusions showed increases in blood pressure of systolic > 30 mmHg and diastolic > 15 mmHg, with none requiring intervention (31).

However, a higher incidence of elevated blood pressures were observed in a retrospective study that reviewed 203 patients with treatment resistant depression who were treated with intravenous ketamine in a clinical setting. Specifically, 44.3% of all patients exhibited transient “treatment-emergent” hypertension, defined as SBP ≥ 165 mmHg or diastolic blood pressure ≥ 100 mmHg at any point during treatment (32). Twelve percentage of these patients were given labetalol or amlodipine to treat hypertension, but the exact indication for treatment was not clear. Of note, no specific events that would meet the criteria for hypertensive emergency were mentioned in this study.

An earlier report on pooled data from 3 studies of 84 participants and 205 total infusions found mean peak blood pressures of systolic 141.9 mmHg and diastolic 86.4 mmHg with blood pressures returning to baseline at 60–70 min after infusion (33). In this analysis, 30% of participants experienced a “clinically significant” increase in blood pressure, defined as blood pressure >180/100 mmHg or heart rate >110 bpm, with no reports of EOD, thus no instances met the criteria for hypertensive emergency. Similarly, review of cardiovascular effects seen in short-term ketamine for TRD found that some trials showed blood pressure levels exceeding 180/100 mmHg, but this was overall quite rare, and again, no serious or persistent cardiovascular events were reported (21).

### Emergency medicine perspective on ketamine associated hypertension

Pre-infusion blood pressure measurement is prudent, but the presence of a hypertensive reading with no signs and symptoms of acute stroke, cardiac ischemia, pulmonary edema, encephalopathy, and congestive heart failure need not be considered an absolute contraindication for treatment. While generally patients receiving non-emergent ketamine treatment should have any pre-existing hypertension treated, patients may commonly have elevations in blood pressure pre-treatment that can even be related to anxiety or white coat hypertension. Recommendations to not proceed with treatment if blood pressure is elevated may be unnecessarily limiting and should be revisited.

Current administration guidelines for ketamine infusions recommend frequent monitoring of vital signs before, during, and after infusion. This is accompanied by a recommendation and suggestions to pause or discontinue infusion, or treat with anti-hypertensives when blood pressures rise above systolic 160 mmHg and/or diastolic 100 mmHg (1), while previously discussed trials utilize a systolic blood pressure ≥ 180 mmHg or diastolic blood pressure ≥ 110 mmHg as a threshold for discontinuing ketamine infusion or initiating treatment with anti-hypertensives. However, in EM these patients would fall under the category of asymptomatic hypertension, in which no medical intervention is recommended.

The intention to treat elevated blood pressure levels during ketamine infusions is well meaning, however, pausing the infusion and/or treating with anti-hypertensives is not without their own adverse effects. Pausing or stopping the infusion because of elevated blood pressure means that the patient may not receive the therapy for their TRD or acute suicidal ideation. It could be argued that leaving either of these conditions untreated also causes an immediate increase in patient morbidity and mortality risk. Additionally, treatment with anti-hypertensives is not benign. A large systematic review examining pharmacologic treatments for hypertensive urgency found that all medications used were associated with side effects. Labetalol, the most frequently used medication to acutely lower blood pressure has been associated with side effects such as dizziness, drowsiness, headache, bradycardia, and pain at the injection site (34). Similar generalized and hemodynamic side effects of anti-hypertensive treatment were seen with other classes of medications such as ACE inhibitors, calcium channel blockers, and vasodilators (34).

Elevated blood pressures that do not meet the criteria for hypertensive emergency should not be treated with antihypertensive agents (18, 35). In addition to side effects of the antihypertensives themselves, they may produce more dangerous hypotensive episodes that could provoke syncope, or worse, if blood pressure is lowered too rapidly in chronically hypertensive patients, stroke or myocardial infarction could occur (36, 37). Routine blood pressure checks mid-infusion offer little benefit and may be distressing to a patient experiencing dissociation. In keeping with the above-described management of hypertension, healthy patients receiving ketamine for depression need not routinely have blood pressure frequently monitored.

Hypertensive emergency has not been documented in IV ketamine trials, so may be considered an extremely rare, though very serious potential adverse event. Rather than monitoring numbers, it is vital for medical personnel administering ketamine infusions to monitor patients for alarming symptoms such as severe chest pressure/pain, acute dyspnea, severe abdominal pain, or significantly decreased (not altered) level of consciousness (that would be defined as a GCS <8). If these occur, blood pressure should be checked. If there is an accompanying hypertensive blood pressure (≥ 180/110 mmHg), anti-hypertensive treatment should be considered with immediate transport to an ED for further workup, management, and evaluation. Anxiety, blurred vision, dizziness, headache, and nausea or vomiting are common ketamine side effects, and not considered signs or symptoms of hypertensive emergencies.

## Discussion and future directions for optimal blood pressure management in IV ketamine treatment

Intravenous ketamine administration is a shift outside the realm of traditional psychiatric treatment for depression. Initial guidelines for patient monitoring reflected the anesthetic nature of this medication and erred on the side of caution given the initial experimental and novel nature of the treatment. However, as efficacy data has accumulated, safety data and clinical experience has as well. The approach to blood pressure could be reconsidered, given the previously described best practices for asymptomatic hypertension management. The EM perspective suggests that for patients who are medically stable with no cardiovascular disease, routine continuous cardiac monitoring and/or frequent vital sign measurements are not necessary and may carry potential harms if blood pressure elevations are treated when asymptomatic. Instead, clinical monitoring for worrisome signs and symptoms (Table 1) could alert to a potential hypertensive emergency and are more essential during IV ketamine treatment. If such symptoms are observed, they would serve as a trigger to check the patient's blood pressure. If elevated, it would prompt consideration for treatment and medical specialist consultation for further workup and management of the presumed hypertensive emergency.

Although a hypertensive emergency has not been reported with IV ketamine, it must also be considered that common practice in psychiatric clinical trials and ketamine programs have followed previous recommendations. This includes intervening by pausing an infusion or treating with antihypertensives when blood pressure rises above a threshold. The practice of responding to an asymptomatic elevation is not consistent with the previously described EM best practice, but it must be considered that this approach has prevented any episodes of hypertensive emergency. Any change in practice to reflect EM best practices must be accompanied by vigilance for warning signs and symptoms that would signal a hypertensive emergency and immediate action must be taken if observed.

Another key issue this perspective raises is eligibility for ketamine therapy. Previous exclusion criteria for IV ketamine cited examples as uncontrolled hypertension, unstable medical condition, central aneurysmal disease, significant valvular disease, recent myocardial infarction, or New York Heart Association Class III heart failure (1, 5). In reconsidering the true risks of hypertension (34) and contrasting these eligibility criteria to those for electroconvulsive therapy - where patients are fully anesthetized - it would seem prudent that these instead be relative contraindications considered on a case by case basis, similar to electroconvulsive therapy. Patients with depression often carry other medical comorbidities, in particular a correlation with increased rates of hypertension (38–40) and generalized cardiovascular disease (41, 42), which may preclude treatment under current criteria. While the aim is for hypertension to be properly treated prior to initiating ketamine treatment, it is not uncommon for physical health to be neglected in severe depression. In considering the EM perspective, an elevated blood pressure alone should not necessarily preclude ketamine treatment. As is common practice for electroconvulsive therapy, the presence of multiple comorbidities could prompt medical consultation prior to initiating a treatment course.

The combined accumulation of experience with ketamine for TRD and the EM perspective provided by this paper should provoke a reconsideration of best practices within psychiatry for intravenous ketamine therapy. Although this paper focused on IV ketamine, addressing hypertension associated with similar treatments including intranasal esketamine or non-IV ketamine should be similarly reconsidered through this EM lens. Protocols must balance the minimal risk with healthy individuals, the variety of comorbidities in the depressed patient, the low rate of complications, and the potential harms of treating transient hypertension if it is not a hypertensive emergency. The goal is an appropriate safety monitoring regimen that will not overburden limited resources and will avoid unnecessary interventions which may carry their own risks. Continued interdisciplinary input, both in protocols and individual consultation for at-risk patients will be important to offer optimal care.

## Data availability statement

The original contributions presented in the study are included in the article/supplementary material, further inquiries can be directed to the corresponding author.

## Author contributions

RY wrote the manuscript draft and was supervised by KS. KS and JS contributed to writing of subsequent drafts. RY and KS provided the emergency medicine context for this article. JS, AK, and RM provided the psychiatry context and discussion. RY, JS, AK, RM, and KS reviewed the paper and provided edits. All authors contributed to the article and approved the submitted version.

## Funding

KS salary was supported by the Canadian Institute of Health Research through a Scientific Director's Grant (SOP 168483) to Dr. Brian Rowe. Funding for the publication costs of this manuscript was provided by the Department of Psychiatry at the University of Alberta.

## Conflict of interest

Author AK has received speaker/consultation fees from Lundbeck, Elvium, Pfizer, Otsuka, Takeda, Sunovion, Bausch Health, Novo Nordisk, Eisai, Jazz, Paladin, the Newly Institute and Abbvie. Author RM has received research grant support from CIHR/GACD/National Natural Science Foundation of China (NSFC) and the Milken Institute; speaker/consultation fees from Lundbeck, Janssen, Alkermes,Neumora Therapeutics, Boehringer Ingelheim, Sage, Biogen, Mitsubishi Tanabe, Purdue, Pfizer, Otsuka, Takeda, Neurocrine, Sunovion, Bausch Health, Axsome, Novo Nordisk, Kris, Sanofi, Eisai, Intra-Cellular, NewBridge Pharmaceuticals, Viatris, Abbvie, Atai Life Sciences and also he is a CEO of Braxia Scientific Corp. Author JS has received speaker/consultation fees from Abbvie, Eisai, Lundbeck, Bausch Health, Janssen, and serves as a medical advisor for the Newly Institute. The remaining authors declare that the research was conducted in the absence of any commercial or financial relationships that could be construed as a potential conflict of interest.

## Publisher's note

All claims expressed in this article are solely those of the authors and do not necessarily represent those of their affiliated organizations, or those of the publisher, the editors and the reviewers. Any product that may be evaluated in this article, or claim that may be made by its manufacturer, is not guaranteed or endorsed by the publisher.
